# Dysregulation of Diverse Ion Transport Pathways Controlling Cell Volume Homoestasis Contribute to Neuroglial Cell Injury Following Ischemic Stroke

**DOI:** 10.1007/s12975-014-0324-3

**Published:** 2014-01-27

**Authors:** Dandan Sun, Kristopher T. Kahle

**Affiliations:** 1 Department of Neurology, University of Pittsburgh School of Medicine, Pittsburgh, PA 15217 USA; 2 Department of Neurosurgery, Massachusetts General Hospital and Harvard Medical, Boston, MA 02115 USA; 3 Manton Center for Orphan Disease Research, Children’s Hospital Boston, Boston, MA 02115 USA

This special featured issue of *Translational Stroke Research* gives readers a timely update on the emerging roles of glutamate receptor-independent ion channels and transporters in ischemic brain injury. This issue has collected contributions from the leading research laboratories with expertise on the most important ion transport targets. For example, Khanna et al. [1] provide an overview on the significance of dysregulated ion homeostasis in the neurogliovascular unit in the pathogenesis of ischemic cerebral edema, focusing on the role of the sulfonylurea receptor 1-transient receptor potential melastatin 4 (SUR1-TRPM4) channel complex which is upregulated in the context of hypoxia and promotes capillary fragmentation and oncotic cell death in the vascular endothelium, thereby increasing vascular permeability and vasogenic edema. Song et al. write an elegant review on the distinct changes of cell volume homeostasis in necrotic, apoptotic, and hybrid cell death that accompany ischemic stroke, along with the *N*-methyl-d-aspartate-independent ion transporters and channels that mediate apoptotic volume decrease or cytotoxic cell swelling [2]. Norenberg et al. [3] report novel mechanisms underlying astrocyte swelling and brain edema mediated by SUR1-TRPM4 activation in acute liver failure, extending original findings by the Simard group in ischemic disease to other types of brain swelling.

The second half of the issue include comprehensive discussions of voltage-gated K^+^ channels [4] and the acid-sensing ion channel 1a ASIC1a [5, 6] in ischemic neurotoxicity, and voltage-gated H^+^ channel Hv1 in microglial activation [7], following stroke. Uria-Avellanal et al. [8] present compelling clinical and experimental research data on overstimulation of Na^+^/H^+^ exchangers and alkaline pHi in perinatal brain injury. Pignataro et al. discuss a strategy of targeting increased Ca^2+^ efflux mediated by Na^+^/Ca^2+^ exchanger in stroke intervention [9]. Chen et al. review the significance of ion channels in regulation of neuronal progenitor function in ischemic brains, which holds the potential for tissue regeneration—an emerging area in the field of neuroprotection [10].

In summary, these timely papers illustrate exciting new findings in the area of glutamate receptor-independent mechanisms of ischemic ionic injury in the nervous system, and reveal the critical role of dysregulated ion transport, mediated by diverse ion transport proteins, in the neurogliovascular unit. These reports show that ischemia-induced ion channel/transporter dysfunction affects multiple critical cell processes, including cell volume regulation [11], intracellular ionic homeostasis [12], neuroinflammation [13, 14], and neuroregeneration/tissue repair [15, 16]. An increased understanding of the role these transport proteins play in normal biology, along with further knowledge of how these proteins are regulated by post-translational modification and signal transduction, might reveal novel methods by which these transport systems may be modulated for therapeutic benefit.

Perhaps nowhere closer is the reality of clinical translation of basic findings more apparent than in the story of the SUR1-regulated TRPM4 ion channel, and its role in the pathogenesis of cerebral edema and hemorrhagic transformation following ischemic stroke (see review by Khanna et al., in this issue). Glibenclamide, an FDA-approved drug commonly used in patients with type 2 diabetes, inhibits SUR1-TRPM4, and several robust preclinical studies have demonstrated the efficacy of glibenclamide in reducing edema and hemorrhagic conversion in rodent models of ischemic stroke. This has in turn prompted the study of the potential protective effects of glibenclamide in humans in an ongoing prospective phase II clinical trial, and preliminary data from this effort suggests glibenclamide also significantly reduces ischemic cerebral edema and hemorrhagic conversion [17]. Many other targets listed above are promising candidates at the cusp of human translational studies. We are excited to present this timely resource to the readers of *Translational Stroke Research*.
